# Supplementary material for: Direct regulation of the voltage sensor of HCN channels by membrane lipid compartmentalization
Source: Nat Commun. 2023 Oct 18;14:6595. doi: 10.1038/s41467-023-42363-7 (PMC10584925; doi:10.1038/s41467-023-42363-7)
Supplement: Supplementary file 3 — Reporting Summary [file 41467_2023_42363_MOESM3_ESM.pdf]

nature portfolio

Corresponding author(s): Gucan Dai

Last updated by author(s): Sep 29, 2023

Reporting Summary

Nature Portfolio wishes to improve the reproducibility of the work that we publish. This form provides structure for consistency and transparency in reporting. For further information on Nature Portfolio policies, see our Editorial Policies and the Editorial Policy Checklist.

Statistics

For all statistical analyses, confirm that the following items are present in the figure legend, table legend, main text, or Methods section.

n/a	Confirmed	
☐	☒	The exact sample size (*n*) for each experimental group/condition, given as a discrete number and unit of measurement
☐	☒	A statement on whether measurements were taken from distinct samples or whether the same sample was measured repeatedly
☐	☒	The statistical test(s) used AND whether they are one- or two-sided<br>*Only common tests should be described solely by name; describe more complex techniques in the Methods section.*
☒	☐	A description of all covariates tested
☒	☐	A description of any assumptions or corrections, such as tests of normality and adjustment for multiple comparisons
☐	☒	A full description of the statistical parameters including central tendency (e.g. means) or other basic estimates (e.g. regression coefficient) AND variation (e.g. standard deviation) or associated estimates of uncertainty (e.g. confidence intervals)
☐	☒	For null hypothesis testing, the test statistic (e.g. *F*, *t*, *r*) with confidence intervals, effect sizes, degrees of freedom and *P* value noted<br>*Give P values as exact values whenever suitable.*
☒	☐	For Bayesian analysis, information on the choice of priors and Markov chain Monte Carlo settings
☒	☐	For hierarchical and complex designs, identification of the appropriate level for tests and full reporting of outcomes
☒	☐	Estimates of effect sizes (e.g. Cohen's *d*, Pearson's *r*), indicating how they were calculated

Our web collection on statistics for biologists contains articles on many of the points above.

Software and code

Policy information about availability of computer code

Data collection	PatchMaster (HEKA Elektronik), INFINITY ANALYZE and CAPTURE (TELEDYNE LUMENERA), VistaVision (ISS, Inc.)
Data analysis	Igor Pro 9, ImageJ 1.49, VistaVision (ISS, Inc.)

For manuscripts utilizing custom algorithms or software that are central to the research but not yet described in published literature, software must be made available to editors and reviewers. We strongly encourage code deposition in a community repository (e.g. GitHub). See the Nature Portfolio guidelines for submitting code & software for further information.

Data

Policy information about availability of data

All manuscripts must include a data availability statement. This statement should provide the following information, where applicable:

- Accession codes, unique identifiers, or web links for publicly available datasets
- A description of any restrictions on data availability
- For clinical datasets or third party data, please ensure that the statement adheres to our policy

Data availability statement is provided with the paper. All Source Data sets for Figs. 1, 2, 3, 4, 5 and S7 are included as Source Data file. Other data are available from the corresponding authors upon request. PDB accession code used in the Fig. S8 has been referred to in the data availability statement.

Research involving human participants, their data, or biological material

Policy information about studies with human participants or human data. See also policy information about sex, gender (identity/presentation), and sexual orientation and race, ethnicity and racism.

Reporting on sex and gender	N/A
Reporting on race, ethnicity, or other socially relevant groupings	N/A
Population characteristics	N/A
Recruitment	N/A
Ethics oversight	N/A

Note that full information on the approval of the study protocol must also be provided in the manuscript.

Field-specific reporting

Please select the one below that is the best fit for your research. If you are not sure, read the appropriate sections before making your selection.

☒ Life sciences ☐ Behavioural & social sciences ☐ Ecological, evolutionary & environmental sciences

For a reference copy of the document with all sections, see nature.com/documents/nr-reporting-summary-flat.pdf

Life sciences study design

All studies must disclose on these points even when the disclosure is negative.

Sample size	Sample size was based on previously published results applying ensemble tmFRET that generated sufficient statistical power, and also with the consideration of technical aspect of applying dual stop-codon suppression for genetic code expansion on imaging membrane ion channels.
Data exclusions	No data were excluded from analysis.
Replication	All experiments were repeated and reproducible. Key experiments were done at least on two different cell transfections. Specific number of independent replicates is noted in the figure legends.
Randomization	The experiments were conducted randomly, i.e. control experiment imaged first and then treatment group, or vice versa
Blinding	The investigators maintained awareness of the experimental conditions throughout the data acquisition and analysis process, and the investigators were unbiased in both outcome assessment and conclusion drawing.

Reporting for specific materials, systems and methods

We require information from authors about some types of materials, experimental systems and methods used in many studies. Here, indicate whether each material, system or method listed is relevant to your study. If you are not sure if a list item applies to your research, read the appropriate section before selecting a response.

Materials & experimental systems

n/a	Involved in the study	
☒	☐	Antibodies
☐	☒	Eukaryotic cell lines
☒	☐	Palaeontology and archaeology
☒	☐	Animals and other organisms
☒	☐	Clinical data
☒	☐	Dual use research of concern
☒	☐	Plants

Methods

n/a	Involved in the study	
☒	☐	ChIP-seq
☒	☐	Flow cytometry
☒	☐	MRI-based neuroimaging

Eukaryotic cell lines

Policy information about cell lines and Sex and Gender in Research

Cell line source(s)	Established tsA 201 cells, embryonal kidney, SV40 transformed, purchased from MilliporeSigma, catalog number 96121229
Authentication	Cell lines were authenticated and confirmed using STR profiling by Millipore Sigma, no further authentication was performed
Mycoplasma contamination	MycoFluor™ Mycoplasma Detection Kit (Invitrogen) was used to test for contamination and the result was negative.
Commonly misidentified lines (See ICLAC register)	No misidentified cell lines. No other cell lines used in the lab.
